# Monitoring the Burden of Seizures and Highly Epileptiform Patterns in Critical Care with a Novel Machine Learning Method

**DOI:** 10.1007/s12028-020-01120-0

**Published:** 2020-10-06

**Authors:** Baharan Kamousi, Suganya Karunakaran, Kapil Gururangan, Matthew Markert, Barbara Decker, Pouya Khankhanian, Laura Mainardi, James Quinn, Raymond Woo, Josef Parvizi

**Affiliations:** 1 Ceribell Inc., 2483 Old Middlefield Way, Suite 120, Mountain View, CA, USA.; 2 Department of Neurology, The Mount Sinai Hospital, New York, NY, USA.; 3 Department of Neurology and Neurological Sciences, Stanford University Medical Center, 300 Pasteur Drive, Stanford, CA 94305, USA.; 4 Department of Neurology, Perelman School of Medicine, University of Pennsylvania, Philadelphia, PA, USA.; 5 Department of Emergency Medicine, Stanford University Medical Center, Stanford, CA, USA.

**Keywords:** Machine learning method, Neurology, Electroencephalography, Status epilepticus, Seizure burden

## Abstract

**Introduction:**

Current electroencephalography (EEG) practice relies on interpretation by expert neurologists, which introduces diagnostic and therapeutic delays that can impact patients’ clinical outcomes. As EEG practice expands, these experts are becoming increasingly limited resources. A highly sensitive and specific automated seizure detection system would streamline practice and expedite appropriate management for patients with possible nonconvulsive seizures. We aimed to test the performance of a recently FDA-cleared machine learning method (Claritγ, Ceribell Inc.) that measures the burden of seizure activity in real time and generates bedside alerts for possible status epilepticus (SE).

**Methods:**

We retrospectively identified adult patients (*n* = 353) who underwent evaluation of possible seizures with Rapid Response EEG system (Rapid-EEG, Ceribell Inc.). Automated detection of seizure activity and seizure burden throughout a recording (calculated as the percentage of ten-second epochs with seizure activity in any 5-min EEG segment) was performed with Claritγ, and various thresholds of seizure burden were tested (≥ 10% indicating 4.5 min of ≥ 30 s of seizure activity in the last 5 min, ≥ 50% indicating ≥ 2.5 min of seizure activity, and ≥ 90% indicating ≥ 4.5 min of seizure activity and triggering a SE alert). The sensitivity and specificity of Claritγ’s real-time seizure burden measurements and SE alerts were compared to the majority consensus of at least two expert neurologists.

**Results:**

Majority consensus of neurologists labeled the 353 EEGs as normal or slow activity (*n* = 249), highly epileptiform patterns (HEP, *n* = 87), or seizures (≥ 90% seizure burden) with 100% sensitivity and 93% specificity. The sensitivity and *n* = 17, nine longer than 5 min (e.g., SE), and eight shorter than 5 min]. The algorithm generated a SE alert (≥ specificity of various thresholds for seizure burden during EEG recordings for detecting patients with seizures were 100% and 82% for ≥ 50% seizure burden and 88% and 60% for ≥ 10% seizure burden. Of the 179 EEG recordings in which the algorithm detected no seizures, seizures were identified by the expert reviewers in only two cases, indicating a negative predictive value of 99%.

**Discussion:**

Claritγ detected SE events with high sensitivity and specificity, and it demonstrated a high negative predictive value for distinguishing nonepileptiform activity from seizure and highly epileptiform activity.

**Conclusions:**

Ruling out seizures accurately in a large proportion of cases can help prevent unnecessary or aggressive over-treatment in critical care settings, where empiric treatment with antiseizure medications is currently prevalent. Claritγ’s high sensitivity for SE and high negative predictive value for cases without epileptiform activity make it a useful tool for triaging treatment and the need for urgent neurological consultation.

## Introduction

The timely diagnosis and treatment of patients with seizures can prevent significant morbidity and mortality [1]. Approximately 30% of patients with altered mental status in critical care settings have seizures, and over 90% of these are nonconvulsive seizures that can only be detected with electroencephalography (EEG) [2–6]. There is rising awareness among healthcare practitioners that the burden of seizures, including nonconvulsive seizures, is associated with brain injury and, thus, continuous or frequent seizures (i.e., a high seizure burden) merit timely detection and treatment [7–10]. For this reason, both timely interpretation of EEG data and timely and accurate quantification of seizure burden are paramount to minimizing brain injury.

Unfortunately, the conventional practice of EEG in critical care and emergency department settings suffers from both delayed access to EEG recordings and significant delays in its interpretation by skilled neurologists [11, 12]. When actionable EEG interpretations are not immediately available, treatment decisions are made on the basis of clinical suspicion alone, which results in the potential for missing or undertreating some patients with nonconvulsive seizures and overtreating a significantly larger number of patients without seizure activity who may not need aggressive antiseizure medications [13, 14]. To provide timely access to EEG, novel rapid EEG systems can be used to enable physicians and allied health professionals to acquire EEG within minutes and stream the data in real time to the cloud, where a machine learning-derived classification algorithm can be applied [15–17].

The use of artificial intelligence (AI) in clinical medicine has been on the rise, and within the specialty of neurology, brain signals have proven particularly amenable to the machine learning approach [18]. There are few software programs commercially available to detect seizures or epileptiform discharges and mark the EEG tracing to help streamline expert review by neurologists. More novel methods for automatic detection of seizures and epileptic spikes have also been described in the literature with various methods and varying degrees of accuracy [19]. Such algorithms are designed to work solely with traditional EEG systems, which as noted are often delayed or unavailable, especially during after-hours and weekends [11, 12] and are too cumbersome for nonneurology experts to use at the bedside. While a full review of these methods is beyond the scope of our present work, it is noteworthy that no method has yet been developed to provide an automated and quantified metric of *seizure burden* (i.e., frequency of seizures per unit of time) to help bedside practitioners caring for critically ill patients. Providing such feedback would allow for risk stratification and evaluation of treatment response, as well as for determining the urgency of requesting neurological consultation, in real time.

The use of AI-assisted programs for EEG interpretation is becoming increasingly necessary as the utilization of EEG is expanding in the fields of critical care and emergency medicine while human resources are scarce, and detailed review of many simultaneous continuous EEG recordings by neurologists in real time is simply too cost prohibitive to be deployed at scale. As a result, there exists a significant unmet need for automated algorithms that could assist nonexperts by providing a reliable *risk stratification tool* using EEG data in real time [18, 20, 21]. Such a tool could alert the bedside nurse or provider on call when it detects a near-continuous epileptiform pattern resembling status epilepticus that may require urgent management and enable providers to see the real-time effect of administered antiseizure medications on the burden of seizure activity.

In the current study, we aim to validate a supervised machine learning algorithm, labeled as Claritγ (Ceribell Inc., Mountain View, California), that was recently approved by the FDA and is being used in clinical settings as a clinical decision support tool. We designed this retrospective study to measure the performance of the algorithm applied to EEG data acquired using Ceribell’s Rapid Response EEG system (Rapid-EEG) from patients in critical care and emergency department settings and to describe its potential clinical implications.

## Methods

### Rapid Response EEG System

**Fig. 1:**
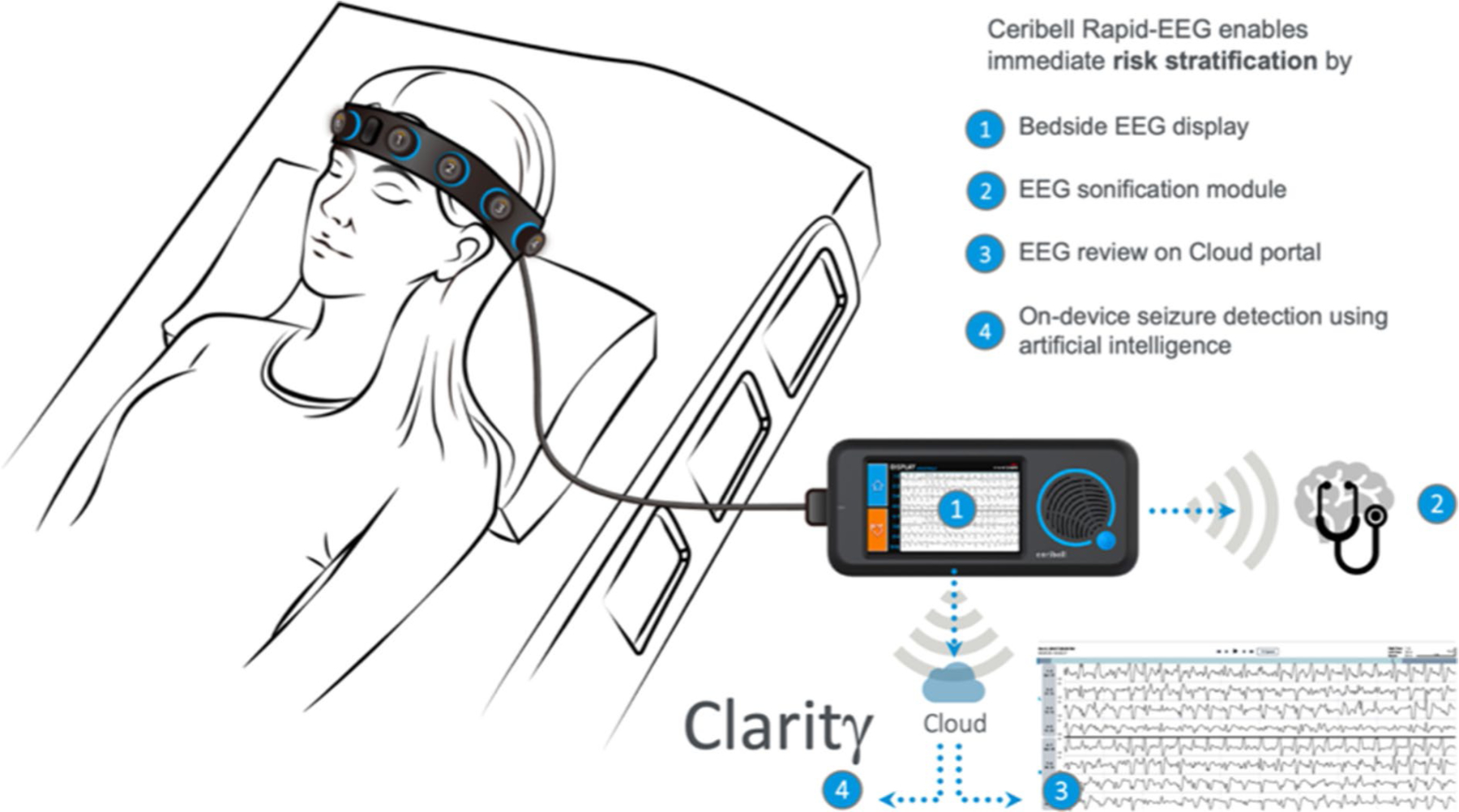
Rapid Response EEG system. The Rapid Response EEG system (Rapid-EEG) consists of a portable EEG recorder and a disposable electrode headband. Recorded EEG tracings are shown on the device screen (1) and sonified when needed (2) by the bedside recorder. HIPAA-compliant secure Wi-Fi connection enables real-time transfer of the data to the cloud where the EEG tracings can be reviewed by expert neurologists using the remote portal for EEG review (3). Machine learning computations (by Claritγ algorithm) are performed on the cloud portal (4) interfacing in real time with the bedside device. As such, the system is meant to provide not only easy and fast access to EEG acquisition, but also a reliable and actionable diagnostic information for risk stratification using four different modes of triage

The Rapid-EEG system (Fig. 1, see www.ceribell.com for additional information) consists of a headband with ten electrodes connected to a handheld recorder. The headband is placed circumferentially around the head and is fastened over the forehead with a locking clasp, and EEG setup is typically performed by nurses, allied health professionals, or healthcare providers trained by either online or in-person sessions. The Rapid-EEG electrodes (1–5 on the left, 6–10 on the right; electrode number increases anterior to posterior, i.e., leads 1 and 6 are near the frontal pole and leads 5 and 10 are near the occiput) correspond approximately to the lateral chains of the International 10–20 system (Fp1–F7, F7–T3, T3– T5, and T5–O1 on the left; Fp2–F8, F8–T4, T4–T6, and T6–O2 on the right), and a longitudinal bipolar montage is constructed to display the EEG waveforms. During EEG recording, the system simultaneously measures the impedance between two adjacent electrodes once per minute using a test frequency that is outside the EEG recording band to avoid creating artifact. The data are acquired as digital samples at a rate of 250 Hz. The handheld recorder displays, records, and wirelessly transmits the data to a remote cloud server, where a cloud-based seizure detection software continuously monitors the EEG recording.

### EEG Data

All EEGs were obtained with Rapid-EEG (Ceribell Inc., Mountain View, California). The recordings were from adult patients (≥ 18 years old) undergoing evaluation for altered mental status and possible seizures in intensive care units and emergency departments at six academic and community hospitals across the USA between January 2018 and April 2019. EEG data were anonymized, and no identifying demographic or clinical information was accessed for this study. The study was classified as exempt research according to the US Department of Health and Human Services regulation 45 CFR 46.104(d) (4), and individual patient consent was not required. It should be noted that the Claritγ algorithm was developed using training and testing datasets that were entirely different and independent from the dataset we have used in the current study for the validation of the algorithm performance.

### Validating Claritγ Seizure Burden Algorithm

**Fig. 2:**
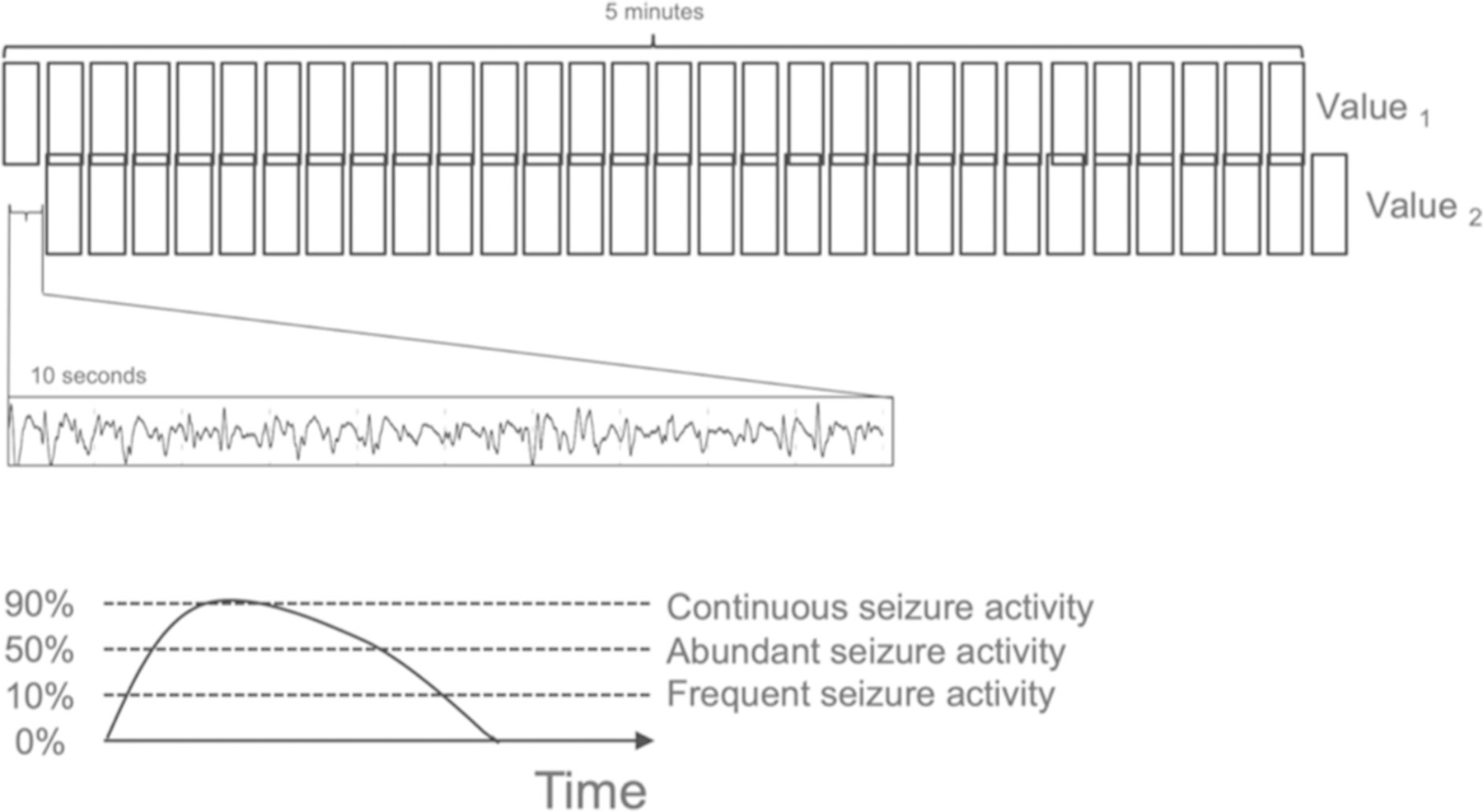
Computation of seizure burden. The output of the Claritγ algorithm was a continuous quantitative trend of seizure burden values, which represented the percentage of 10-second long bins of EEG data in a 5-min period that contained seizure activity. Seizure burden values updated every 10 s; therefore, consecutive seizure burden values (e.g., value 1 and 2, as shown, offset by 10 s) could represent the evolution of the patient’s seizure prevalence over the course of the recording. Seizure burden thresholds were adapted from American Clinical Neurophysiology Society guidelines [22], such that “frequent” seizure activity was defined as 10% seizure burden (i.e., 30 s of seizure activity within a 5-min period), “abundant” seizure activity was defined as 50% seizure burden (i.e., 2.5 min of seizure activity within a 5-min period), and “continuous” seizure activity was defined as 90% seizure burden (i.e., 4.5 min of seizure activity within a 5-min period). An alert was presented to the user when seizure burden reached a threshold of 90%, which indicated a high risk of status epilepticus and the impending need for urgent clinical intervention

We validated the performance of the Claritγ algorithm in a cohort of 353 Rapid-EEG recordings from 353 patients. An overview of the algorithm is shown in Fig. 2. The signal from each EEG channel was filtered and segmented into nonoverlapping ten-second bins. Time-domain and frequency-domain features were calculated for each ten-second bin of EEG signal. Seizure activity was defined for each ten-second bin using multiple features, including measures of power (power within each frequency band and their ratios), morphology (signal amplitude, variability, distribution, and change over time), rhythmicity and regularity (measures of entropy), and correlation (cross-channel correlation of signals). For each ten-second bin, the algorithm classified the segment of signal as either seizure or nonseizure in a deterministic (rather than probabilistic) manner. Seizure burden was calculated as the percentage of ten-second bins of EEG data in a 5-min period that were classified as seizure activity. This seizure burden value is updated every ten seconds to generate a rolling 5-min window, resulting in a continuous seizure burden trend that represents the evolution of the patient’s seizure prevalence over the course of monitoring. The maximum seizure burden for the duration of each recording was used as the final output of the algorithm for this study; however in clinical practice, the seizure burden value and trend would be available continuously.

Seizure burden, the prevalence of seizure activity within any 5-min period, was described using thresholds adapted from American Clinical Neurophysiology Society guidelines [22] as follows: 10% was defined as “frequent” (indicating 30 s of seizure activity), 50% was defined as “abundant” (indicating 2.5 min of seizure activity), and 90% was defined as “continuous” (indicating 4.5 min of seizure activity). A seizure burden of 90% (4.5 min) indicates activity approaching the definition of status epilepticus (5 min), so the algorithm would present an alert to the user at any point when the seizure burden reaches a threshold of 90%.

### Reference Standard Defined by Expert Neurologists’ Review of EEG

**Fig. 3:**
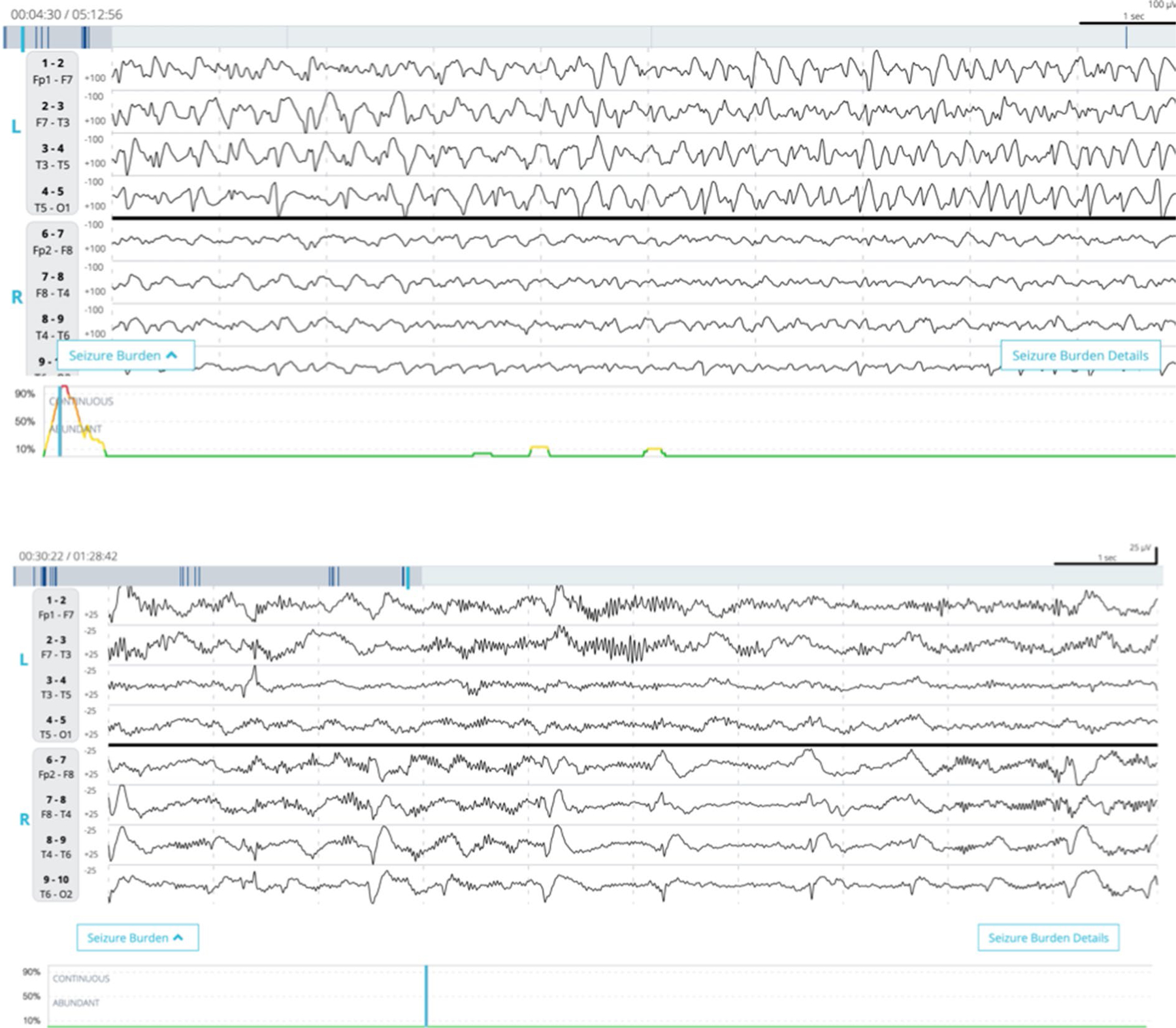
Samples of EEG recorded with Ceribell Rapid Response EEG System. Each EEG is displayed in a ten-second epoch with filter settings of 1–30 Hz. The line plot under each EEG shows the Claritγ algorithm output. The top image shows seizure activity approaching the 90% threshold to trigger a status epilepticus alert, and the bottom image shows lateralized periodic discharges that go undetected by the algorithm

Each EEG file was independently reviewed by at least two independent neurologists with fellowship training in clinical neurophysiology or epilepsy. Reviewers were blinded to patients’ clinical information, including medical history, indication for EEG monitoring, prior treatment with antiseizure medication, and Claritγ seizure burden trend. Expert consensus (reference standard) was defined by agreement between a minimum of two neurologists; additional reviewers were consulted if the first two neurologists did not agree until a majority consensus was reached. Reviewers were instructed to indicate whether the EEG contained normal, diffusely slow, highly epileptiform, or seizure activity. Highly epileptiform patterns (HEP) included activity that did not fully meet the Salzburg criteria [23] for electrographic seizure activity, but did represent abnormal electrographic epileptiform activity such as periodic discharges or lateralized rhythmic delta activity [24, 25]. These patterns merited separate consideration from other nonseizure patterns for two major reasons. First, these patterns exist on a spectrum of pathologic activity along with seizures—the so-called ictal–interictal continuum (IIC)—and at times, they can be difficult to distinguish from seizures, especially absent ancillary clinical information [26–28]. Second, these patterns might benefit from treatment with antiseizure medications, and measuring the performance of the algorithm in these cases would have clinical significance [26, 29–31]. See Fig. 3 for a representative sample of each of these categories recorded using Rapid-EEG. Each EEG recording and each individual labeled episode were divided into one of three categories based on the most severe pattern present in the recording defined by expert majority consensus: seizures (high severity), HEP (intermediate severity), and normal or slow activity (low severity). Therefore, a reference standard was generated for individual episodes as well as for the overall EEG recording. EEG readers also labeled the start and end of the EEG patterns, which allowed for calculation of seizure duration that could be compared to the algorithm output.

### Statistical Analysis

We defined the reference (“gold”) standard as the consensus agreement of at least two neurologists reading the same EEG, both for each overall EEG record and for individual episodes of expert-identified seizures. We then tabulated Claritγ algorithm output of seizure burden against this reference standard. Using these tabulations, we calculated the sensitivity, specificity, and false detection rate (number of false positive events divided by the total duration of EEG recordings in hours) of various seizure burden thresholds (10%, 50%, 90%) against the expert consensus; 95% CIs for sensitivity and specificity measures were calculated using established formulas [32]. It should be noted that the three thresholds are cumulative—i.e., all 90% alerts by definition generate both a 10% and a 50% notification, and all 50% alerts by definition first generate a 10% notification. Our validation study was not designed to optimize among the three different thresholds.

Given the wealth of evidence [33–36] describing the variability between expert EEG reviewers, whether due to human error or to differences in interpretation, we sought to contextualize the observed diagnostic accuracy of the seizure detection algorithm by quantifying the inter-rater variability. We calculated the sensitivity and specificity of identifying EEGs with status epilepticus for each of the experts that reviewed at least 250 h of EEG. No single expert reviewed all of the cases, and because each of the experts reviewed a different subset of the recordings, we could not quantify inter-rater variability using Cohen’s *κ*.

## Results

### Reference Standard

A total of 353 Rapid-EEG recordings were evaluated in this study with a cumulative EEG recording duration of 1052 h (mean EEG duration: 233 ± 227 min). No cases were excluded. Expert consensus determined the most severe feature of the overall EEG record to be status epilepticus in nine cases, seizures (duration < 4.5 min) in eight cases, HEP in 87 cases, and normal or low background in 249 cases (Table 1). Within the 353 recordings, a total of 47 discrete seizure events were identified by expert consensus.

### Algorithm Performance

Claritγ algorithm output of seizure burden compared against expert consensus diagnosis for both overall EEG records and individual events is summarized in Table 1, and performance (in terms of sensitivity, specificity, and false detection rate) is summarized in Table 2. Claritγ detected ≥ 90% seizure burden (seizure activity ≥ 4.5 min, thereby triggering an alert for impending status epilepticus) in nine out of nine Rapid-EEGs with status epilepticus, and within these cases, Claritγ correctly detected ≥ 90% seizure burden in 12 out of 13 discrete events of seizure lasting ≥ 4.5 min. Therefore, the sensitivity for identifying status epilepticus was 100% for the overall record, and the sensitivity for identifying individual seizure episodes that were ≥ 4.5 min was 92.3%.

The specificity of the ≥ 90% seizure burden notification was 93.0%, resulting in a false detection for only 24 out of 353 EEGs. In 21 (87.5%) of these false detections, the expert consensus categorization of the EEG was HEP, indicating that there was concerning epileptiform activity present in the record, even if no unequivocal seizures were agreed to be present by both reviewers.

Claritγ correctly identified 41 of the 47 total discrete seizure events of any duration (88.2% sensitivity). In the 1052 h of cumulative EEG across the 353 EEG recordings, Claritγ had 383 false detections, resulting in an overall false detection rate of 0.36 per hour. Of the 179 EEG recordings in which Claritγ detected no seizures, seizures were identified by the expert reviewers in only two cases (negative predictive value of 99%). In both cases, the seizures missed by Claritγ were less than 30 s in duration.

The four experts reviewed at least 250 h of the overall dataset (1052 total hours). When these four reviewers’ identifications of status epilepticus were compared to the majority consensus (Table 3), individual expert raters displayed variability in both sensitivity (range 20–89%) and specificity (range 94–99%). In general, reviewers with the lowest sensitivity also had the highest specificity, a natural trade-off. It is also important to note that, in some cases, reviewers classified a status epilepticus case as a highly epileptiform pattern (HEP). This was considered a miss and reduced the reviewer’s sensitivity even though the reviewer did not consider the EEG to be normal. Each expert reviewed a different subset of the 353 total recordings. Between the four experts, a total of 784 individual reviews were performed (an average of 2.22 reviews per EEG recording). No single expert reviewed all of the cases. The number of reviews performed by each expert is shown in Table 3. Overall, experts displayed greater specificity compared to Claritγ, and each of the reviewers misclassified at least one case of status epilepticus.

## Discussion

**Fig. 4:**
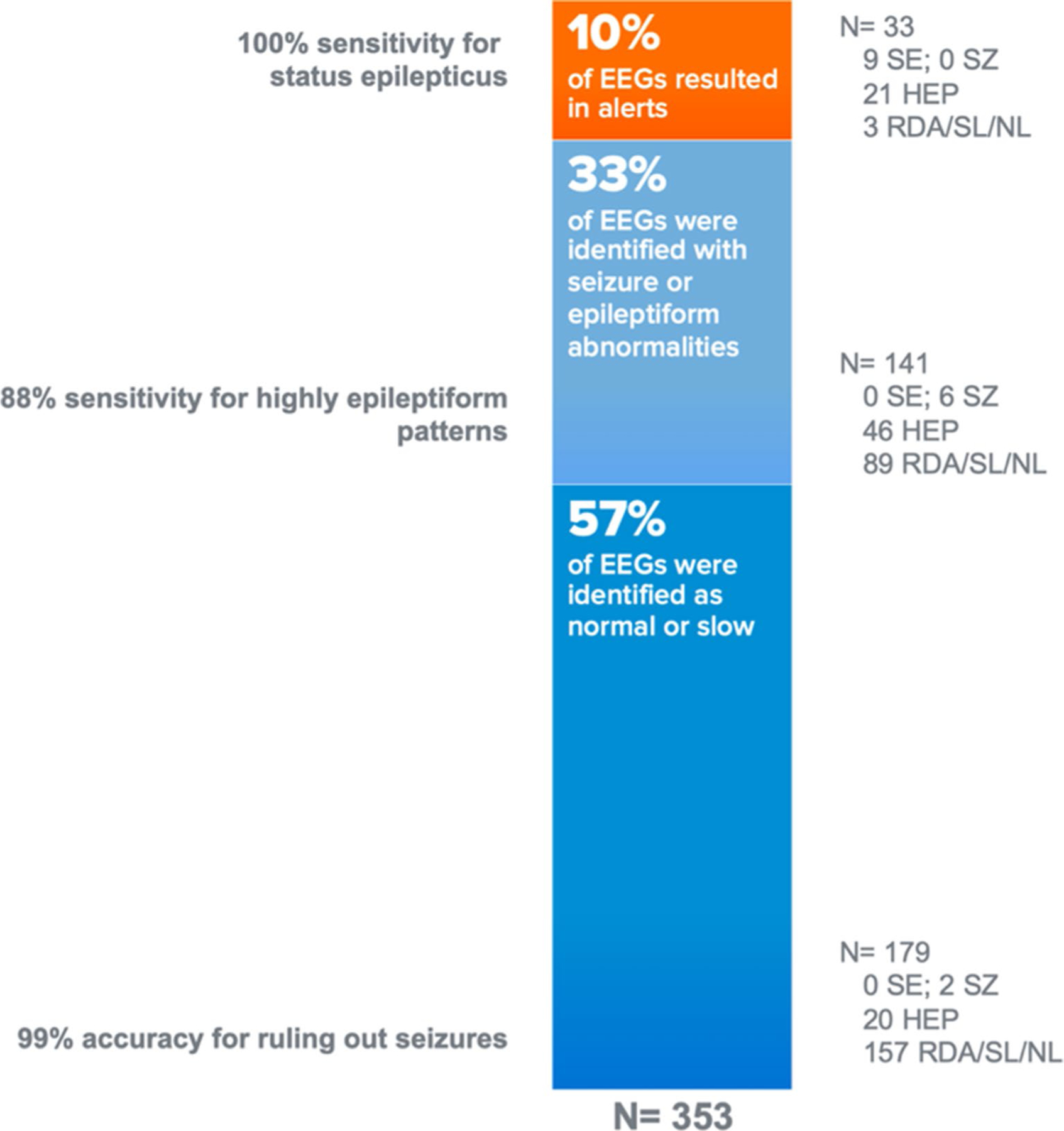
Summary of Claritγ Performance. Performance of Claritγ algorithm at the group level suggests that the algorithm can be seen as a reliable triage tool to help detect cases of status epilepticus with the highest sensitivity (while overcalling about one-fourth of highly epileptiform patterns as possible status epilepticus). It also performs as a reliable triage tool to help physicians avoid over-aggressive treatments in majority of EEG cases where the overwhelming pattern is either slowing or normal. *HEP* highly epileptiform patterns, *NL* normal activity, *RDA* rhythmic delta activity, *SE* status epilepticus, *SL* slow activity, *SZ* seizure

In this study, we describe the development of an artificial intelligence machine learning algorithm for *seizure burden measurement* using EEG data acquired with Rapid Response EEG. This algorithm showed high sensitivity (100%) in the detection of status epilepticus (even outperforming some neurologists with fellowship training in clinical neurophysiology or epilepsy) and accurately identified 88% of seizures of any duration when compared to the reference standard of consensus of expert neurologists (Fig. 4). The specificity of the algorithm for seizures at all seizure burden thresholds was principally limited by “misclassification” of highly epileptiform patterns. These patterns possess multiple “seizure-like” qualities trained into our algorithm (e.g., rhythmicity, sharply contoured morphology, high amplitude, and extant correlation across channels) as seizures. Raising the seizure burden threshold to trigger an alarm (i.e., only alerting the user if the burden reaches 90% level) increased the algorithm’s specificity for prolonged seizures as high as 93%. As such, the algorithm performance can be considered to be reliable and valid at the extremes of the spectrum of seizure burden (i.e., possible status epilepticus and normal diffusely slow activity), but considerably less so if the burden of abnormality is less frequent or brief in duration. While our new algorithm output provides a trendline and a means to assist in triage/prioritization, it is important for users to understand its lower specificity for milder cases of abnormality or shorter duration seizures.

### Suggested Clinical Implementation Workflow

In considering the potential clinical implications of Rapid Response EEG with Claritγ, it is important to ensure its proper integration into existing workflows to secure its maximum (and safe) impact on physicians’ clinical decision making and patient management. We remind the reader that the output of any AI algorithm ought to be interpreted in the context of the user’s pretest clinical judgment. In keeping with this, we have made an initial attempt to recommend a possible workflow for the use of our algorithm in the current clinical practice of Rapid-EEG (Table 4). We are mindful that the workflow may need to be modified in different settings depending on the resources available and the clinical division where the Rapid-EEG technology is being utilized.

As suggested in this workflow, Claritγ algorithm has potential utility as the first of its kind *risk stratification tool* to streamline the practice of stat EEG and guide emergent triage and more precise treatment for patients with low or high suspicion for nonconvulsive seizures. Moreover, it also can lessen the stress and unnecessary burden on neurologists with EEG expertise. We are hopeful that simplified risk stratification offered by our algorithm combined with the earlier and easier acquisition of EEG will lead to better management of patients in need.

### Study Limitations

The lack of clinical data provided to reviewers during their retrospective EEG review was the most important limitation of this study and should be considered highly relevant to the interpretation of its findings. In clinical practice, patient history (e.g., an established diagnosis of epilepsy with a known seizure semiology, recent antiseizure medications) and bedside or video observations (e.g., twitching or behavioral changes) are used to inform EEG interpretation and make definitive determinations of seizure versus nonseizure, and treatment is tailored to the patient as a whole rather than the EEG in isolation. Claritγ, like all other automated seizure detection algorithms, does not consider ancillary clinical information, and it is critical to acknowledge that output from such an algorithm does not provide the final diagnostic conclusion for the patient. Indeed, the sensitivity and specificity of any system for EEG review (human or machine) would be significantly improved by access to additional patient data.

The implementation of Claritγ and its impact on real-world diagnostic and treatment decisions were not studied here and may be addressed in future investigations. As noted in the discussion of clinical workflow, individual providers or practice groups may utilize different alarm thresholds to expedite EEG review and treatment or establish treatment protocols that rely on the various functions of Rapid-EEG (visual waveform review, EEG sonification, Claritγ seizure burden) based on providers’ comfort with each.

Our cohort contained a relatively low number of status epilepticus cases (nine out of 353 EEGs) which affects the reliability of our sensitivity estimate. A larger cohort of Rapid-EEG data would be helpful to address this. However, it should be noted that this multisite cohort was obtained without excluding cases and could be considered reflective of the population being studied.

The Rapid-EEG system lacks midline and parasagittal electrodes, and consequently a focal seizure that is highly localized to the parasagittal region would not be detected by Rapid-EEG or identified by Claritγ (although prior research [37–39] has argued that this lack of coverage might not significantly impact its sensitivity in critical care and emergency medicine settings).

## Conclusions

Implementation of artificial intelligence tools in the field of neurology and epileptology remains limited despite the urgent need for tools to accommodate the expanding practice of EEG and address the inefficiencies of the current EEG infrastructure. Claritγ provides highly sensitive detection of status epilepticus and may be useful as a risk stratification tool that could expedite diagnosis and treatment of patients with nonconvulsive seizures. We believe that machine learning tools will never replace a careful history and examination filtered through a well-honed clinical acumen of the user and recommend its safe use by interpreting its output in the context of proper clinical judgment.

## Figures and Tables

**Table 1:** Summary of Claritγ Performance (individual patient level)

Claritγ output, % seizure burden	Human expert rating, n				
	SE	SZ	HEP	NL/SL	Total
SZ burden ≥ 90%	9	0	21	3	33
SZ burden 50–89%	0	3	21	15	39
SZ burden 10–49%	0	3	20	56	79
SZ burden 1–9%	0	0	5	18	23
SZ burden 0%	0	2	20	157	179
Total	9	8	87	249	353

*HEP* highly epileptiform pattern, *NL* normal background activity, *SE* status epilepticus, *SL* slow background activity, *SZ* seizure

**Table 2:** Sensitivity and specificity of Clarit γ algorithm for seizure detection

Claritγ output	Patient level			Event level			
	*N*	Sensitivity (95% CI)	Specificity (95% CI)	*N*	Sensitivity (95% CI)	False positives (95% CI)	FDR^a^
SZ burden ≥ 90%	9	100.0%^c^	93.00%	13	92.3%^b^	62	0.06
95% CI			[90, 95]		[60, 100]		
SZ burden ≥ 50%	12	100.0%^c^	82.40%	18	100.0%^c^	139	0.13
95% CI			[78, 87]				
SZ burden ≥ 10%	17	88.20%	59.50%	35	80.00%	324	0.31
95% CI		[65, 100]	[54, 65]		[63, 91]		

*CI* confidence interval, *FDR* false detection rate

a False detection rate (in events per hour of EEG) was calculated as the number of false positive events divided by the duration of recording (in hours)

b One seizure event that did not trigger a status alarm occurred during the last 10 min of a 200-min EEG record. The algorithm correctly identified the seizure, but the threshold for 90% seizure burden (4.5 min) was not yet reached at the time the recording was discontinued

c Confidence intervals are not calculated in cases where the sampled sensitivity was 100% as estimated confidence intervals in the event of perfect sample sensitivity do not provide meaningful information

**Table 3:** Variability in status epilepticus detection between individual experts and Claritγ status alert compared to expert consensus

Reviewer	Sessions reviewed	Sensitivity (%)	Specificity (%)
1	240	62.5	94.8
2	198	20.0	99.5
3	257	88.9	94.3
4	89	66.7	95.2
Claritγ	353	100.0	93.0

**Table 4:** Suggested clinical implementation workflow of Claritγ algorithm

		Pretest clinical suspicion	
		High	Low
AI output	> 90%	Treat urgently → urgent review of EEG^a^	Review of EEG → treat if seizures confirmed on EEG or if EEG reading is not readily possible
	10–90%	Review of EEG → treat if seizures confirmed on EEG or if EEG reading is not readily possible	Do not treat yet → Review of EEG whenever possible
	< 10%	Do not treat yet→ Review of EEG whenever possible	Do not treat → nonurgent review of EEG

a Expedited review of EEG can be done remotely in real time by a neurologist with EEG expertise since the Rapid-EEG device sends the EEG data wirelessly to a cloud portal. EEG review can also be performed at the bedside by both expert or nonexpert users. For instance, similar to common models of electrocardiographic monitoring [40] or bedside quantitative EEG products [41, 42], critical care staff may be trained to recognize the most salient and clinically important EEG signatures associated with status epilepticus. They can rely on their own bedside visual EEG review combined with the Rapid-EEG’s *Brain Stethoscope* function [43]. In three clinical studies so far, staff with minimal or no EEG experience increased their accuracy of seizure diagnosis significantly by relying on either *Brain Stethoscope* alone [15, 16] or combined with bedside visual EEG review [17]. It is important to note that a much larger number of EEG cases often do not result in seizure output, and hence, use of the algorithm will lead to prevention of overtreatment of these cases. Given the high sensitivity of the algorithm, false negative cases would be much less frequent
